# Erratum

**DOI:** 10.4269/ajtmh.924err

**Published:** 2015-04-01

In *Am J Trop Med Hyg 89:* 570–577 by Fernandes Cota and others, there is an error in the tables. The authors found a mistake categorization of gender in the study database, which resulted in presentation of an inverted proportion between male and female patients, thereby generating an error in Tables 1 and 2 of the published study. The correct Tables appear here. The authors and the journal regret this error.

## Figures and Tables

**Table 1:** Demographic and clinical characteristics of 113 HIV-infected patients who underwent diagnostic testing for VL

Variable	Patients
Sex (male:female)	76:37
Age, mean years + SD	40,42 ± 10.22
Previous VL (%)	24/113 (21.2%)
CD4 count, median (25–75% interquartile range)*	67 (37–164) cell/mm^3^
PCR-HIV-1 load, median (25–75% interquartile range)†	34.176 (79–184.370) copies/mm^3^
ARV therapy use (%)	70/113 (61.9%)
Fever more than 14 days (%)	81/113 (71.7%)
Cytopenia (%)	111/113 (98.2%)
Splenomegaly (%)	50/113 (44.2%)
Opportunist infection previously (%)	67/113 (59.3%)

SD = standard deviation; VL = visceral leishmaniasis; PCR-HIV-1 = HIV load by real time polymerase chain reaction; ARV = anti-retroviral.

* Available in 95 patients.

† Available in 69 patients.

**Table 2:** Clinical and laboratorial characteristics of patients with target condition (visceral leishmaniasis) present or absent according to adjudication committee definition

	Target condition present, n = 46	Target condition absent, n = 67	*P* value
Age (mean ± SD)	41.02 ± 10.8	40.0 ± 9.8	0.60
Sex (male:female)	37:9	39:28	0.01
Anti-HVC presence	2 (4.3%)	6 (9.5%)	0.44
HbsAg presence	0	2 (3.2%)	0.31
Illicit drug use	16 (34.8%)	27 (40.3%)	0.58
Alcohol abuse	31 (67.4%)	40 (59.7%)	0.39
Previous VL diagnosis	20 (43.4%)	4 (5.9%)	0.00
Previous opportunist infection	31 (67.4%)	36 (53.7%)	0.17
Previous schistosomiasis diagnosis	3 (6.5%)	7 (10.4%)	0.05
Comorbidities	13 (28.3%)	16 (23.8%)	0.64
CD4+ T cell count (25–75% IR)	67 (6–864)*	92 (5–483)†	0.18
HIV-PCR load copies/mm^3^ (25–75% IR)	5.000 (0–65.712)‡	78.132 (7.412–316.650)§	0.01
Fever	28 (60.8%)	53 (79.1%)	0.06
Cytopenia	46 (100%)	65 (97.0%)	0.51
Splenomegaly on physical exam	35 (76%)	15 (22.4%)	0.00
Death in 30 days	4 (8.7%)	8 (11.9%)	0.02
ARV therapy use	32 (69.6%)	34 (50.7)	0.15
ARV therapy regular use	15/32 (46.8%)	7/34 (20.6)	0.01
Hemoglobin g/dl	8.2 ± 1.6	9.1 ± 2.4	0.04
Leucocytes count cell/L (25–75% IR)	2.0 (1.75–2.80) × 10^9^	3.3 (1.75–4.20) × 10^9^	0.005
Platelets count cell/L (25–75% IR)	114 (82.75–173.75) ×10^9^	139 (90.50–244.50) ×10^9^	0.233
rK39 dipstick test positivity	21/46 (45.6%)	2/67 (3.0%)	0.00
IFAT positivity	28/46 (60.9%)	7/67 (10.4%)	0.00
DAT-LPC positivity	41/46 (89%)	8/59 (13.6%)	0.00
*Leishmania* qPCR in peripheral blood positivity	36/42 (85.7%)	4/60 (6.7%)	0.00
Median copies *Leishmania* qPCR in blood (25–75% IR)	16.543 (108–70.209)∥	66.436 (5066–36.479.000)¶	0.56

SD = standard deviation; Anti-HVC = antibody to the hepatitis C virus; HbsAg = hepatitis B surface antigen; ARV = anti-retroviral; PCR-HIV = real time polymerase chain reaction for HIV-1; qPCR = real time polymerase chain reaction for *Leishmania infantum.*

* Performed in 43 patients.

† Performed in 52 patients.

‡ Performed in 29 patients.

§ Performed in 40 patients.

∥ Available in 35 patients.

¶ Available in 4 patients.

